# Green-engineered multispectral analytical approaches for simultaneous quantification of amoxicillin and diclofenac in pure and combined forms

**DOI:** 10.1186/s13065-025-01545-8

**Published:** 2025-06-19

**Authors:** Neamat T. Barakat, Amina M. El-Brashy, Mona E. Fathy

**Affiliations:** Department of Pharmaceutical Analytical Chemistry, Faculty of Pharmacy, Mansoura University, Mansoura, 35516 Egypt

**Keywords:** Amoxicillin, Diclofenac, Spectrophotometry, Univariate methods, Derivative based methods, Ratio-spectra based methods, Greenness

## Abstract

In response to the increasing demand for sustainable and high-throughput analytical approaches, this study presents the development and validation of five innovative univariate spectrophotometric methods for the simultaneous determination of amoxicillin trihydrate and diclofenac sodium in both synthetic mixtures and combined pharmaceutical dosage forms. The proposed methods, comprising first derivative, second derivative, ratio difference, ratio derivative, and ratio subtraction techniques, were designed to address the challenge of overlapping UV spectra, offering enhanced selectivity without the need for complex instrumentation or prior separation. Each method was meticulously optimized and validated in accordance with ICH Q2(R1) guidelines, demonstrating excellent linearity, accuracy, precision, and robustness. The analytical performance affirms their applicability for routine quality control of the studied drugs in a variety of formulations. Moreover, the environmental footprint of the methods was rigorously assessed using three complementary greenness evaluation tools: the GAPI, NEMI and Analytical eco scale, all of which confirmed the eco-friendly nature of the protocols. These results position the developed methods as a cost-effective, rapid, and green alternative for quality assurance in pharmaceutical laboratories, striking a valuable balance between analytical reliability and environmental responsibility.

## Introduction

Simultaneous determination of two or more drugs using the same procedures in one reaction system, as spectrophotometric methods, is one of the interests of the modern analytical chemistry. Such methods are considered to be economic and safe. Since they save cost and time effectively and reduce the consumption of hazardous chemicals. Moreover, spectrophotometric methods achieve more success in resolving the mixture of two drugs specially drugs with overlapping spectra [1]. All these features are realized in this article by applying five simultaneous spectrophotometric methods to resolve this valuable mixture; amoxicillin and diclofenac. The importance of this binary mixture is that it contains an antibiotic and an anti-inflammatory and in one product as in commercial Bipen® capsules. This drug combination offers a novel feature to medical specialists as they can prescribe multi comprehensive treatment in one medication for their patients.

**Fig. 1:**
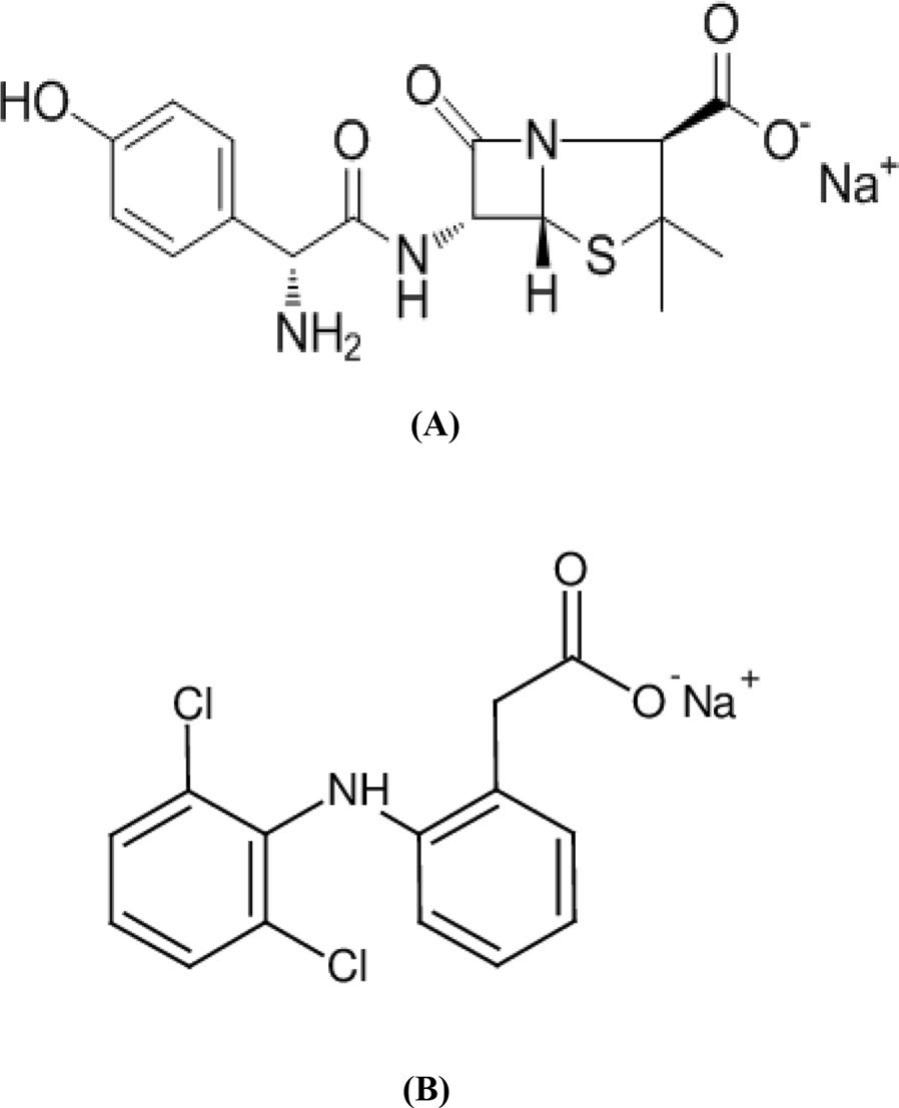
**A** AMC and **B** DLF

Amoxicillin sodium (AMC) is chemically defined as; sodium salt of (2S,5R,6R)-6-[[(2R)-2-Amino(4-hydroxyphenyl) acetyl] amino]-3,3-dimethyl-7-oxo-4-thia-1-azabicyclo [3.2.0] heptane-2-carboxylic acid [2], Fig. 1A. It is a broad spectrum an antibiotic used in the treatment of infections caused by gram-positive and gram-negative bacteria, especially tonsillitis, dental abscess, osteomyelitis and upper respiratory tract infections [3]. Due to its wide use and its importance, various analytical methods have been reported for assay of AMC either alone or combined with other drugs as; spectrophotometry [4–10], chromatography [11], spectrofluorimetry [12, 13], capillary electrophoresis [14] and electrochemistry [15–18].

Diclofenac sodium (DLF) is chemically defined as; sodium salt of 2-[(2,6-Dichlorophenyl) amino] benzene acetic acid [2]. Figure 1B. It is a one of non-steroidal anti-inflammatory drugs used for the relief of pain and inflammation in various conditions: musculoskeletal and joint disorders such as rheumatoid arthritis, osteoarthritis and ankylosing spondylitis, soft-tissue disorders such as sprains and strains and other painful conditions such as renal colic, acute gout, migraine and after some surgical procedures [3]. From the literature review, several methods were reported for analysis of DLF including; different chromatographic techniques [19], spectrophotometry [19], spectrofluorimetry [20–23], potentiometry [21] and voltammetry [24, 25].

AMC and DLF are official in the United States Pharmacopoeia (USP) [26] and British Pharmacopoeia (BP) [27]. Furthermore, only two approaches have been reported for their simultaneous estimation [28, 29] which include no univariate spectrophotometric methods have been documented yet. Therefore, the aim of this research is the development of five simple, green, sensitive, accurate and precise spectrophotometric univariate methods for simultaneous analysis of both drugs in their pure forms, synthetic mixture, single and combined dosage forms. These five univariate methods, namely; first derivative (D^1^), second derivative (D^2^), ratio difference (RD), ratio derivative (DD^1^) and ratio subtraction (RS) methods.

## Experimental

### Devices

- Double beam, Shimadzu UV–Vis spectrophotometer with 3.7. software version and 1 cm path length matched quartz cuvettes was used to perform spectrophotometric scan (Kyoto, Japan).
- SS 101H 230 ultrasonic bath (produced by Witeg in Germany).

### Software

Spectrophotometric univariate methods were performed using UV probe software version 2.42. (Kyoto, Japan).

### Materials

#### Raw materials and solvent

- Methanol was supplied from Fisher Scientific UK, Loughborough, Leics (UK).
- AMC with a purity of; 99.82 ± 1.45%, determined by applying the reported HPLC method [29], was kindly provided by Egyptian International Pharmaceutical Industries Company (E.I.P.I.CO.), Cairo, Egypt.
- DLF with a purity of; 100.41 ± 1.69%, determined by applying the reported HPLC method [29] was kindly provided by the National Organization for Drug Control and Research (NODCAR), Cairo, Egypt.

#### Pharmaceutical dosage forms

- E-Mox® capsules, claimed to have 500 mg AMC / capsules, were produced by Egyptian International Pharmaceutical Industries Company (E.I.P.I.CO.), Cairo, Egypt.
- Declofenac® tablets, claimed to contain 50 mg DLF /tablet, were manufactured by elnasr company for pharmaceutical and chemical Industries, Cairo, Egypt.
- Bipen® capsules, assumed to contain 250 mg of AMC and 250 mg of DLF / capsule, were manufactured by Stalwart Remedies Pvt. Ltd, Vadodara, Gujarat, India.

### Standard solutions

AMC and DLF standard solutions were prepared by separately weighing 10 mg of the respective drug into 50.0 mL volumetric flask. Methanol was used to dissolve and dilute these drugs to the mark for obtaining a final concentration of 200.0 µg/mL for each drug. Both solutions remained stable for about one month in the refrigerator.

### Procedures

#### Calibration curves

Different aliquots from standard solutions of both drugs were individually transferred into two different sets of 10.0 mL measuring flasks. Then, further dilution using methanol were performed to obtain working solutions covering final concentration range; 5.0 to 40.0 µg/mL for each drug. Subsequently, these final solutions were spectrophotometrically scanned against blank methanol and their zero order absorption spectra (^0^D) were stored at computer for further processing treatment.

##### First derivative method (D^1^)

The first derivative (D^1^) of the recorded zero-order absorption spectra for amoxicillin trihydrate (AMC) and diclofenac sodium (DLF) was computed using a wavelength increment (Δλ) of 4.0 nm and a scaling factor of 10.0. For the selective quantification of AMC, the D^1^ amplitudes were measured at 219.4 nm, which corresponds to the zero-crossing point of DLF, thereby eliminating its spectral interference. Conversely, DLF was quantified at 316.0 nm, the zero-crossing wavelength of AMC, ensuring exclusive measurement. Subsequently, calibration curves were constructed by plotting the D^1^ peak amplitudes of each drug against their corresponding concentrations. The resulting calibration graphs demonstrated excellent linearity, and the associated regression equations were derived and used for the quantitative analysis of the studied drugs in both pure and combined dosage forms.

##### Second derivative method (D^2^)

The second derivative (D^2^) technique was employed on the zero-order spectra of both drugs, using a scaling factor of 10.0 and a Δλ of 16.0 nm. The AMC spectrum revealed a peak at 231.4 nm, which corresponds to the zero-crossing of DLF, while the D^2^ peak for DLF was observed at 321.2 nm, corresponding to the zero-crossing of AMC. The amplitude values of the derivative peaks for each drug were then plotted against their respective concentrations to construct calibration curves and derive the corresponding regression equations.

##### Ratio spectra difference method (RD)

This approach was carried out by dividing the zero-order absorption spectrum of AMC by the zero-order spectrum of DLF at 10.0 µg/mL, and similarly, dividing the zero-order spectrum of DLF by the zero-order spectrum of AMC at 10.0 µg/mL. The differences in absorbance at 236.6 nm and 245.6 nm for AMC, as well as at 239.4 nm and 294.8 nm for DLF, were then plotted against the concentrations of the respective drugs. Calibration curves were constructed from these plots, leading to the derivation of the corresponding regression equations.

##### Ratio derivative method (DD^1^)

To execute this method, the first derivative transformation with a Δλ of 16.0 nm and a scaling factor of 10.0 was applied to the previously recorded ratio spectra of AMC and DLF. The peak amplitudes were measured at 226.2 nm for AMC and at 284.2 nm for DLF. These amplitudes were then plotted against the respective drug concentrations, allowing the construction of precise calibration curves and the formulation of the corresponding regression equations.

##### Ratio subtraction method (RS)

DLF can be directly quantified by measuring the intensities of its zero-order spectrum at 291.2 nm and plotting these values against its corresponding concentrations. This enables the construction of a calibration curve, from which the regression equation is derived. AMC can be selectively determined by first eliminating the spectral contribution of DLF through division of its zero-order spectrum by a preselected DLF spectrum (20.0 µg/mL). The resulting ratio spectra were then refined by subtracting a constant absorbance value to normalize the baseline. These corrected spectra were subsequently multiplied by the same DLF divisor (20.0 µg/mL) to restore the original scale. Absorbance values at 232.4 nm were then recorded and plotted against the corresponding AMC concentrations to construct the calibration curve and derive the regression equation.

#### Analysis of synthetic mixtures

Various volumes of AMC and DLF standard solutions, covering their ratio (1:1), were transferred into a set of 10.0 mL volumetric flasks and diluted to the volume using methanol. Subsequently, prepared mixtures were scanned from 200.0 to 400.0 nm and the obtained spectra were recorded at computer. Subsequently, the procedures under section “2.5.1.” were applied on ^0^D spectra of the synthetic mixtures and the correlated regression equations were then computed.

#### Analysis of AMC and DLF in their single pharmaceutical dosage forms

For AMC; Ten E-Mox® capsules were weighed, evacuated and their contents were mixed well. Then, an amount of the mixed powder equivalent to 20.0 mg AMC was dissolved and completed to the volume using methanol in 100.0 mL volumetric flask and sonicated for about 30.0 min then filtered.

For DLF; Ten Declofenac® tablets were weighed, finely triturated and mixed well. After that, an accurate weight from the mixed powder equivalent to 20 mg of DLF was quantitively transferred into a 100 mL measuring flask and the flask was completed to the mark using methanol and sonicated for about 30.0 min then filtered.

Different aliquots of AMC and DLF dosage form stock solutions were transferred separately into two different sets of 10.0 mL volumetric flask. Further dilution using methanol was performed to prepare the working solutions. Furthermore, these prepared solutions were scanned in the range from 200.0 to 400.0 nm and the produced ^0^D spectra were recorded at computer. Finally, the procedures of univariate methods represented under section “2.5.1.” were adopted. From the derived regression equations, each drug amount in dosage form was obtained.

#### Analysis of AMC and DLF in their combined commercial capsules

Ten Bipen® capsules (250 mg of AMC and 250 mg of DLF / capsule) were evacuated, weighed and their content were mixed well. A certain weight equivalent to 20 mg of both drugs was quantitively transferred into 100.0 mL volumetric flask, diluted to the mark using methanol, sonicated for about 30.0 min and filtered. Different volumes from this filterate were transferred into a set of 10 mL volumetric flasks and diluted with methanol. Subsequently, the procedures for the five univariate methods represented under section “2.5.1.” were applied and each drug amount was obtained from the corresponding regression equation.

## Results and discussion

**Fig. 2:**
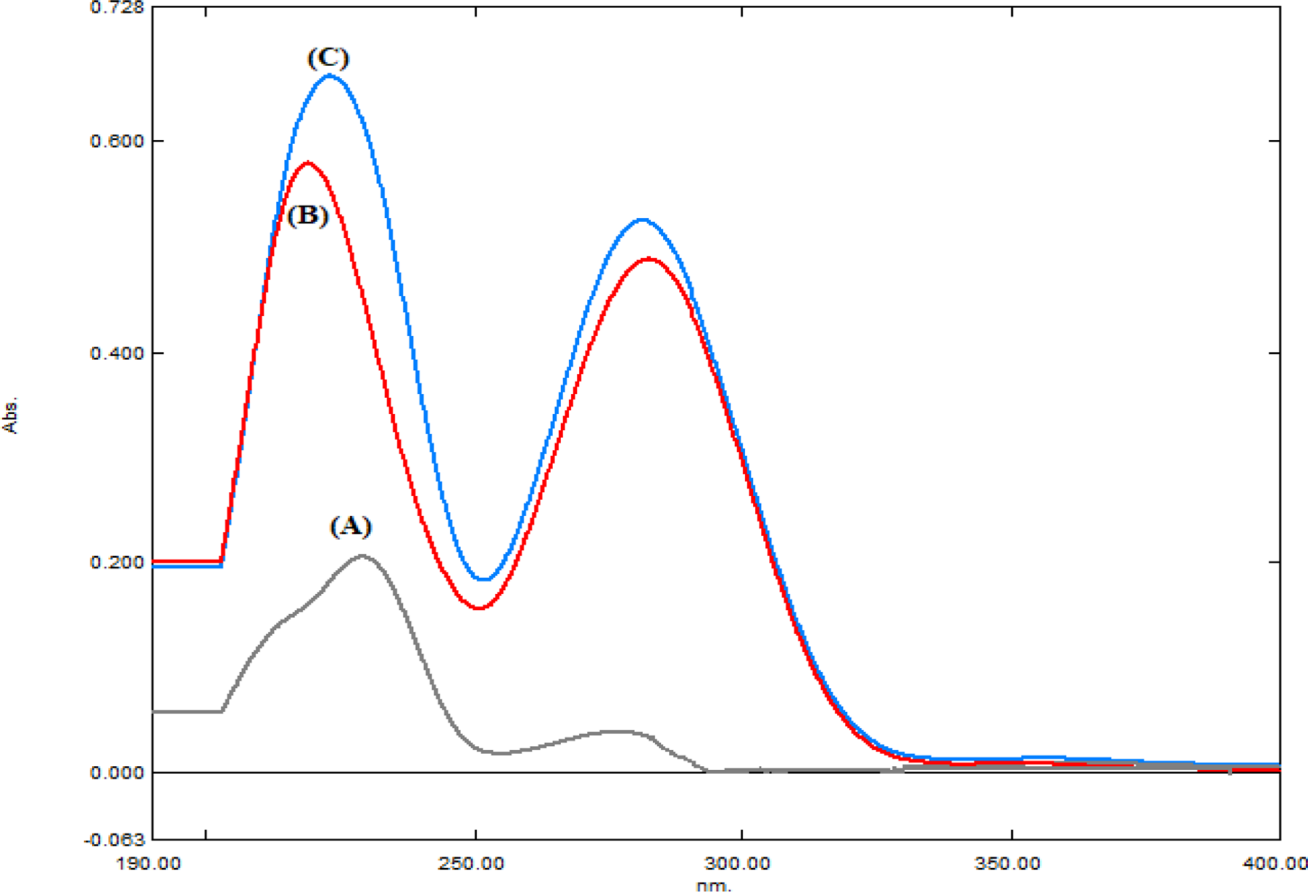
Zero order spectra of: **A** AMC (10.0 µg/mL) and **B** DLF (10.0 µg/mL) **C** synthetic mixture of AMC and DLF (10.0 µg/mL each)

Simultaneous estimation of co-formulated binary mixtures in the field of analytical chemistry has a great importance as it reduces the analyst effort and time which may be needed to analyze each drug separately. In the present study, AMC and DLF were determined simultaneously in their synthetic mixtures and combined capsules without prior separation. Moreover, spectrophotometric methods are the most widely applied ones due to their availability and simplicity compared to other complicated analytical techniques. The chosen spectrophotometric methods for simultaneous assay of the studied binary mixture with highly overlapped zero order spectra (Fig. 2) were investigated, validated according to ICH guidelines [30] and tested regarding their environmental impact.

### Derivative spectrophotometric methods

**Fig. 3:**
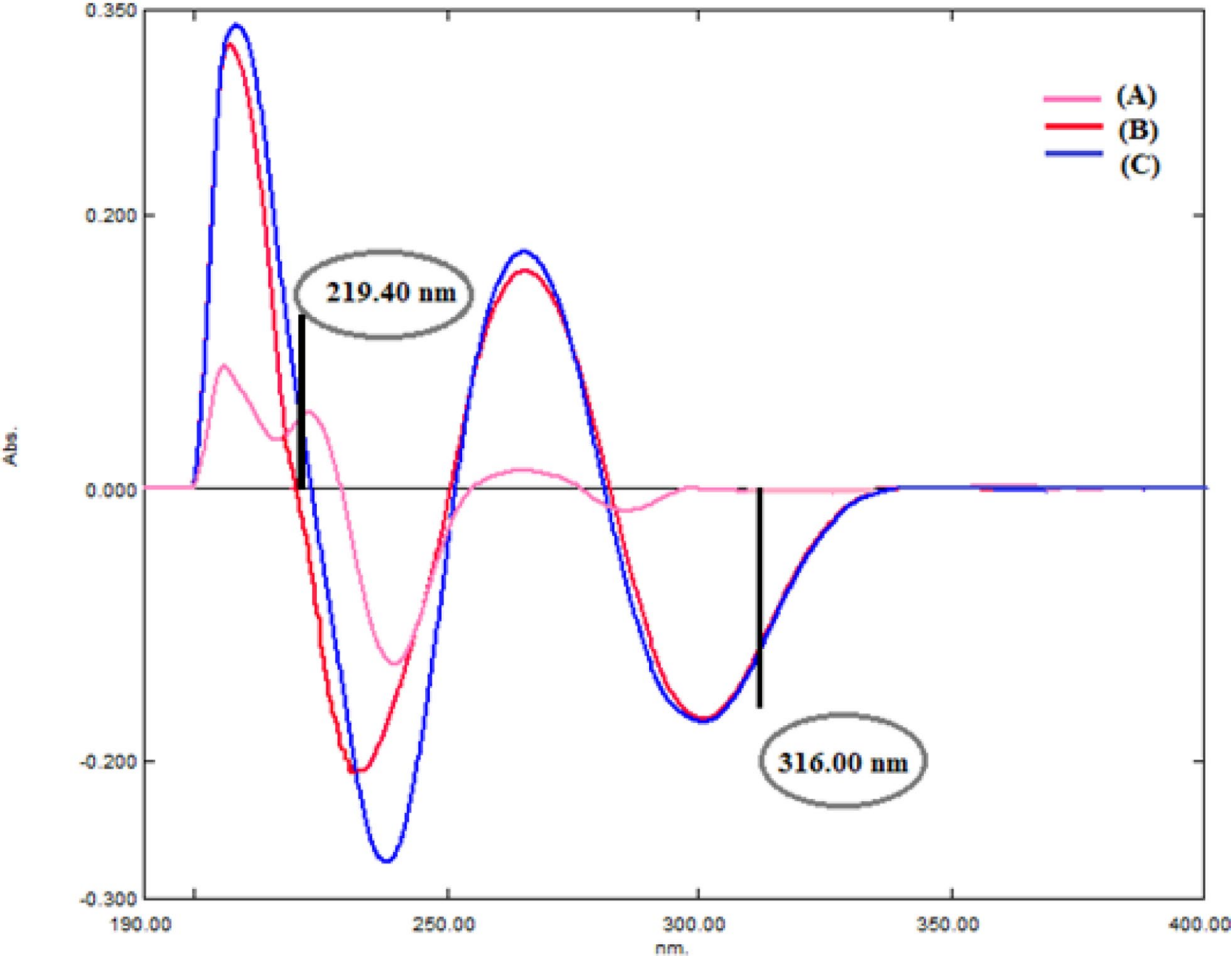
First derivative spectra of **A** AMC (10.0 µg/mL) and **B** DLF (10.0 µg/mL) **C** synthetic mixture of AMC and DLF (10.0 µg/mL each)

**Fig. 4:**
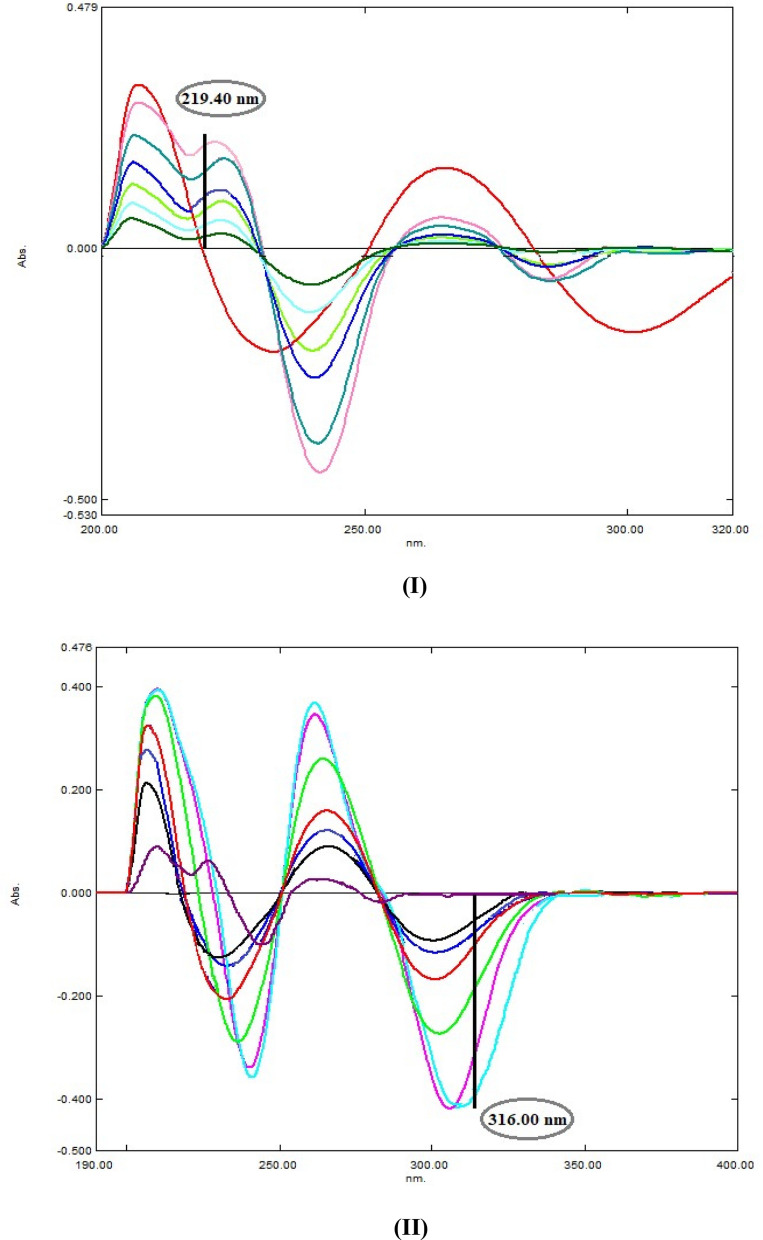
**I** First derivative spectra of AMC (5.0, 10.0, 20.0, 30.0,40.0 µg /mL) at 219.4 nm. DLF (10.0 µg/mL). **II** First derivative spectra of AMC (10.0 µg/mL), DLF (5.0,10.0,20.0,30.0,40.0 µg /mL) at 316.0 nm

Derivative spectroscopy uses first or higher derivatives of absorbance with respect to wavelength for qualitative analysis and for quantification. The concept of derivatizing spectral data was first introduced in the 1950s, when it was shown to have many advantages [31]. It is more accurate and rapid method for resolving and quantification of multiple component mixtures [32, 33]. By applying 1st derivative on both drugs recorded zero order spectra, AMC could be determined by measuring the (D^1^) amplitudes at 219.4 nm (zero crossing point of DLF), while D^1^ amplitudes for DLF were recorded at 316.0 nm (zero crossing of AMC) as shown in Fig. 3. The main instrumental factors, smoothing and scaling factor, which affect the shape of the derivative spectra [34] were studied and optimized. Various smoothing and scaling factors were tried and it was found that Δλ of 4.0 nm and scaling factor 10.0 gave the best results. Then, calibration curves were constructed by graphing (D^1^) amplitudes of each drug versus its final concentrations and regression equations were obtained (Table 1) and (Fig. 4).

**Table 1:** Regression and validation parameters for determination of AMC and DLF by the proposed univariate spectrophotometric methods

Parameter	D^1^		D^2^		RD		DD^1^		RS	
	AMC	DLF	AMC	DLF	AMC	DLF	AMC	DLF	AMC	DLF
λ (nm)	219.4	316.0	231.4	321.2	ΔP 236.6–245.6	ΔP 239.4–294.8	226.2	284.2	232.4	291.2
Range (μg/mL)	5.0–40.0									
Slope	0.0037	0.0107	0.0011	0.0005	0.0194	1.287	0.0149	0.5124	0.0129	0.0285
Intercept	0.0110	− 0.0159	0.0012	0.0006	0.0487	9.585	− 0.0032	− 1.342	0.0575	0.1087
SD of slope (S_b_)	7 × 10^–5^	11 × 10^–5^	1 × 10^–5^	1 × 10^–5^	2.8 × 10^–4^	1.78 × 10^–2^	2 × 10^–4^	5.4 × 10^–3^	1.4 × 10^–4^	2.7 × 10^–4^
SD of intercept (S_a_)	1.5 × 10^–3^	2.6 × 10^–3^	2 × 10^–4^	9 × 10^–5^	6.6 × 10^–3^	0.4152	0.0047	0.0895	0.0033	0.0061
(r)	0.9996	0.9998	0.9998	0.9998	0.9996	0.9996	0.9996	0.9998	0.9998	0.9998
S.D	1.43	1.10	1.38	1.07	1.54	1.24	1.27	1.40	1.37	1.24
% RSD	1.42	1.10	1.38	1.07	1.54	1.24	1.27	1.40	1.38	1.23
% Error	0.58	0.45	0.56	0.43	0.62	0.50	0.52	0.57	0.56	0.51
LOD (μg/mL)	1.355	0.801	0.708	0.568	1.124	1.064	1.054	0.576	0.855	0.716
LOQ (μg/mL)	4.107	2.427	2.146	1.704	3.406	3.225	3.195	1.747	2.593	2.171

**Fig. 5:**
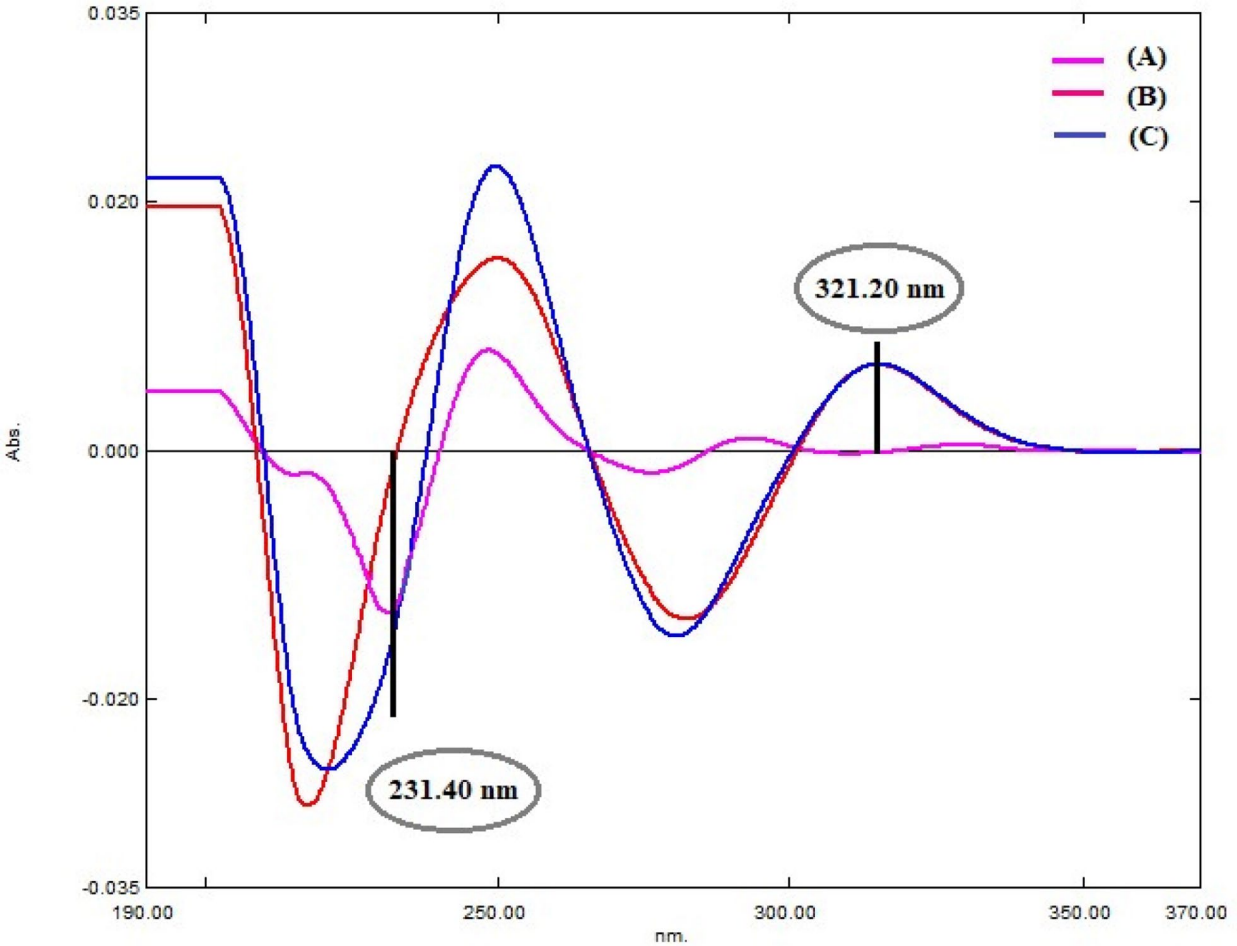
Second derivative spectra of **A** AMC (10.0 µg/mL) and **B** DLF (10.0 µg/mL) **C** synthetic mixture of AMC and DFL (10 µg/mL each)

**Fig. 6:**
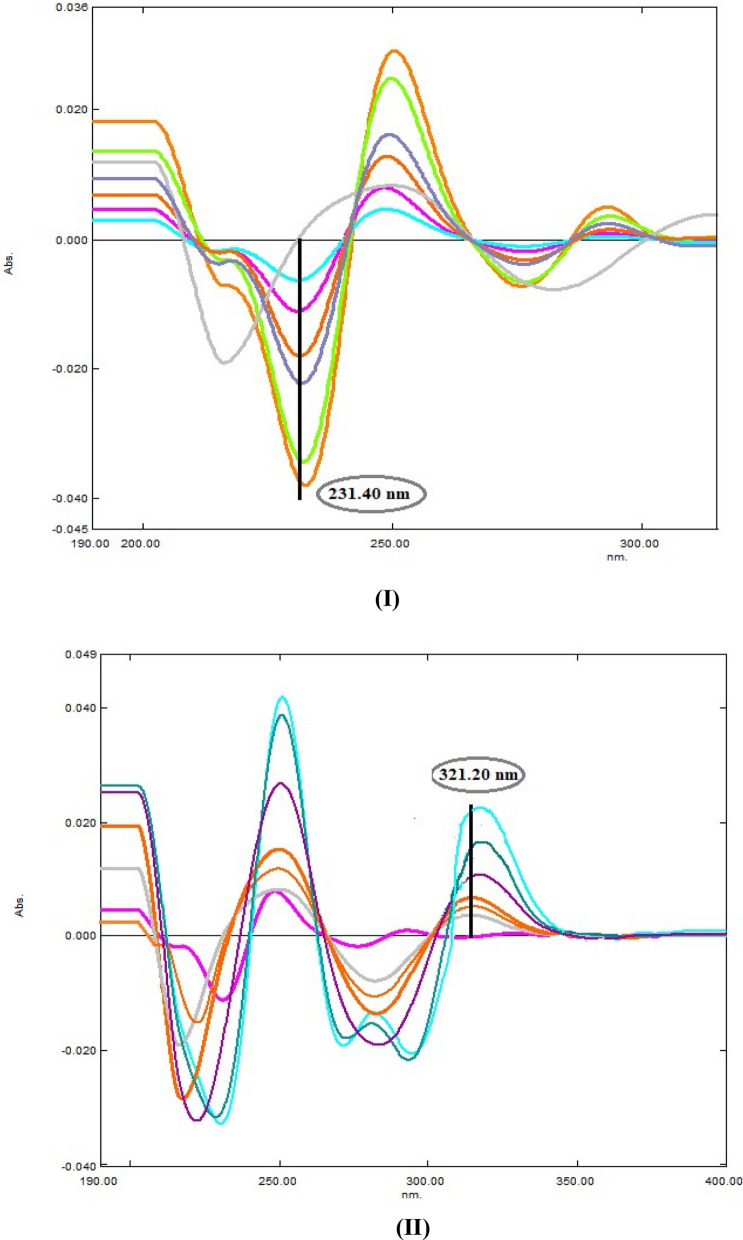
**I** Second derivative spectra of AMC (5.0,10.0,20.0,30.0,40.0 µg /mL) at 231.4 nm. DLF (10.0 µg/mL). **II** Second derivative spectra of AMC (10.0 µg/mL), DLF (5.0,10.0,20.0,30.0,40.0 µg /mL) at 321.2 nm

Furthermore, 2nd derivative method was applied on the recorded zero spectra of both drugs with 16.0 nm smoothing factor and scaling 10.0. The D^2^ peak of AMC were measured at 231.4 nm, zero crossing of DLF, while for DLF quantification; peak amplitudes were recorded at 321.2 nm, zero crossing of AMC (Fig. 5). Subsequently, second derivative calibration curves were obtained by plotting their amplitudes against their final concentrations and the correlated regression equations were deduced (Table 1) and (Fig. 6).

### Ratio spectra-based methods

**Fig. 7:**
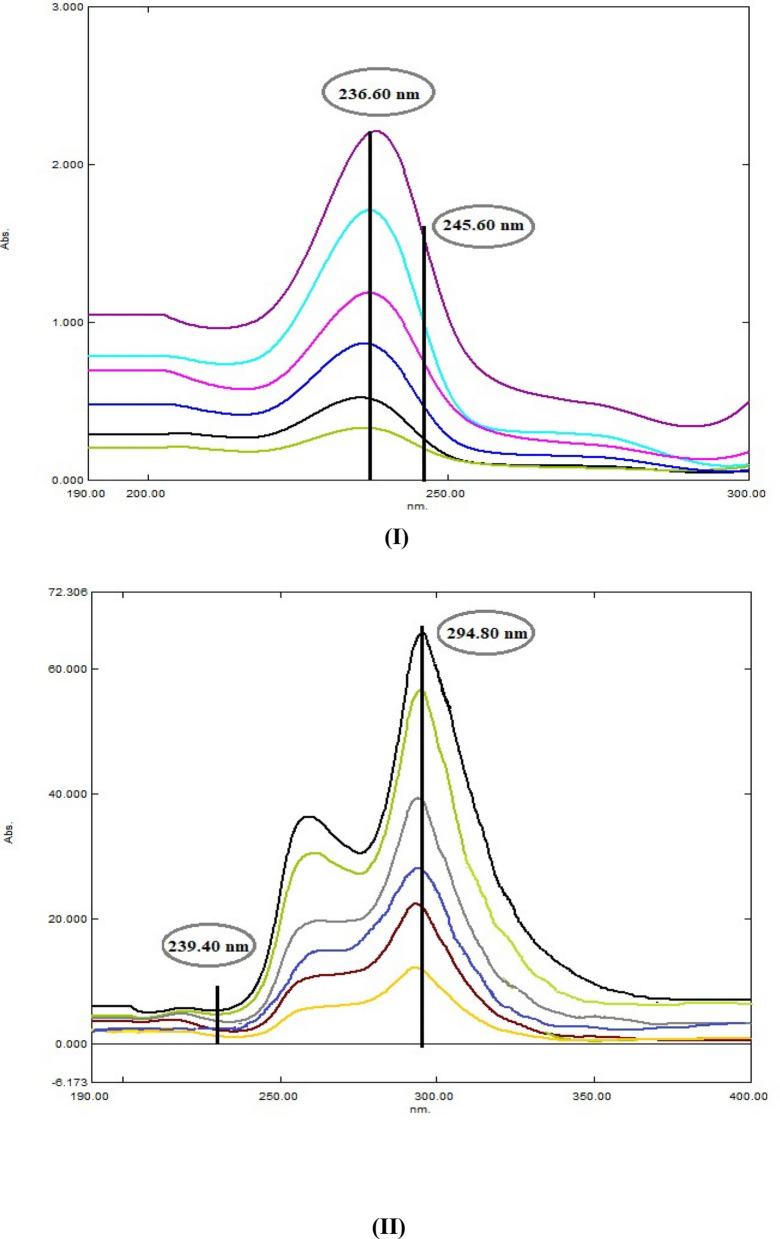
**I** Ratio spectra of AMC in the range of 5.0–40.0 µg/mL using 10.0 µg/mL DLF as a divisor. **II** Ratio spectra of DLF in the range of 5.0- 40.0 µg/mL using 10.0 µg/mL AMC as a divisor

Cancellation of the effect of the whole spectrum of the interfering drug by these suggested approaches is the best key benefit which facilitates simultaneous determination of both drugs. These ratio-based methods were mainly optimized by choosing the divisor carefully to get the best results [35]. Based on the better sensitivity and repeatability and after several trials, the divisors were finally chosen, 10.0 μg/mL of AMC or DLF were used as divisor for DLF or AMC, respectively. The resulted ratio spectra of AMC and DLF in the range of 5.0–40.0 μg/mL are represented in Fig. 7 I and II.

#### Ratio difference spectrophotometric method (RD)

The most striking features of this newly introduced method are its accuracy, rapidity, simplicity and its ability to resolve extremely overlapped spectra without previous separation. furthermore, it doesn’t need any sophisticated computer programs or apparatus. For estimation of the drug of interest by this method, the only requirement is the contribution of the two components at the two carefully selected wavelengths λ 1 and λ 2 where the component of interest shows remarkable difference in the amplitude values and the interfering component ratio spectrum shows constant (equal) amplitudes. Likewise, another two wavelengths were chosen for the assay of the other drug [36]. From AMC and DLF ratio spectra (Fig. 7 I, II), the difference between the absorbance values at (236.6 nm and 245.6 nm) and (239.4 nm and 294.8 nm) were calculated for both drugs, respectively and plotted against corresponding concentrations to obtain their calibration curves and corresponding regression equations (Table 1).

#### Ratio derivative method (DD^1^)

**Fig. 8:**
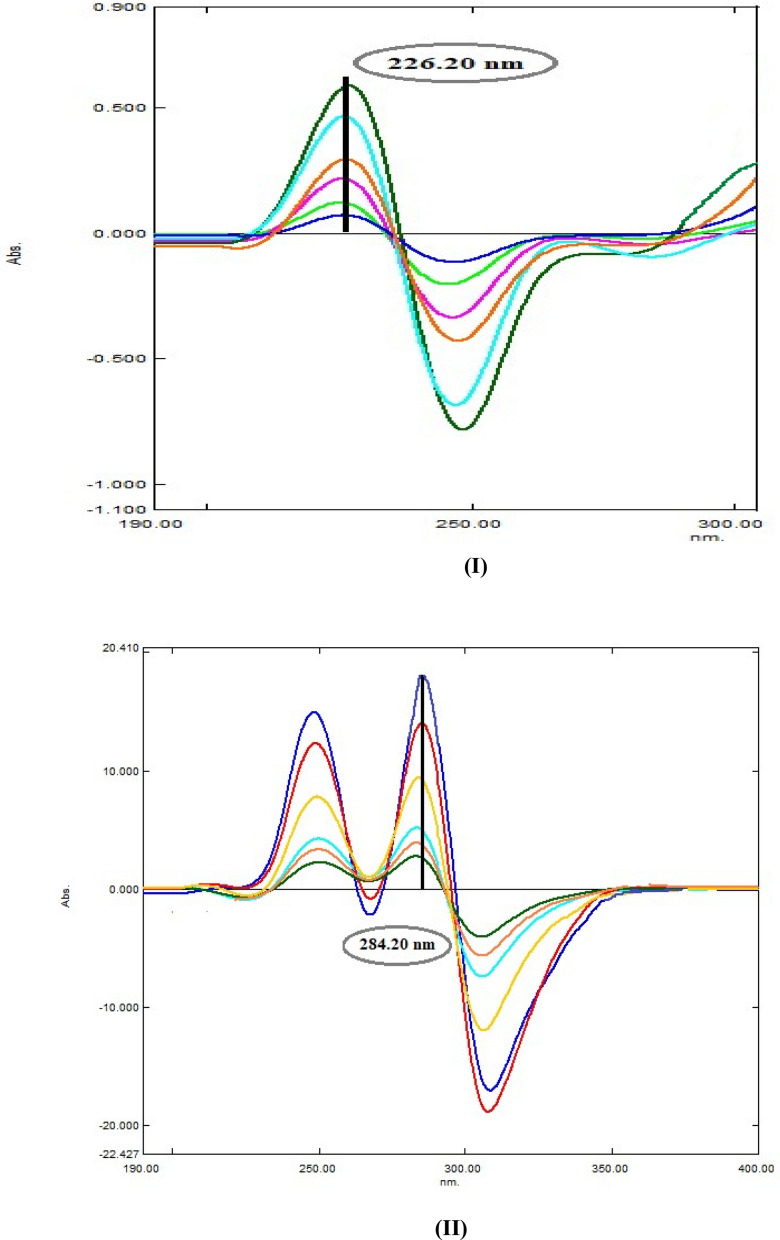
**I** First derivative ratio spectra of AMC in the range of 5.0–40.0 µg/mL using 10.0 µg/mL DLF as a divisor. **II** First derivative ratio spectra of DLF in the range of 5.0–40.0 µg/mL using 10.0 µg/mL AMC as a divisor

This approach was developed by Salinas et al. [37] It can be applied when the first derivative of the previously obtained ratio spectra of both drugs was traced with the Δλ 16.0 nm and scaling factor 10.0 after studying the effect of different Δλ and scaling factors. For AMC, the first derivative of its obtained ratio spectra (DD^**1**^) was illustrated in (Fig. 8 I). and it can be quantified by measuring the absorbance values at 226.2 nm. where there was no effect from DLF. Similarly, wavelength 284.2 nm was chosen for estimation of DLF where there was no interference from DLF, as shown in (Fig. 8 II). Then, the recorded peaks amplitudes of the obtained DD^**1**^ spectra were plotted against each drug corresponding concentrations to obtain their calibration curves and the corresponding regression equations were derived and summarized in Table 1.

#### Ratio subtraction method (RS)

This method was introduced by El-Bardicy et al. [38]. It was efficiently applied in this study. When the D^0^ spectrum of DLF is more extended than AMC spectrum as shown in Fig. 2, the determination of DLF can be done by directly measuring its absorbance values at 291.2 nm in D^0^ spectrum without any interference from AMC. These obtained values were then plotted against corresponding concentrations to construct calibration curve and the regression equation was derived (Table 1).

**Fig. 9:**
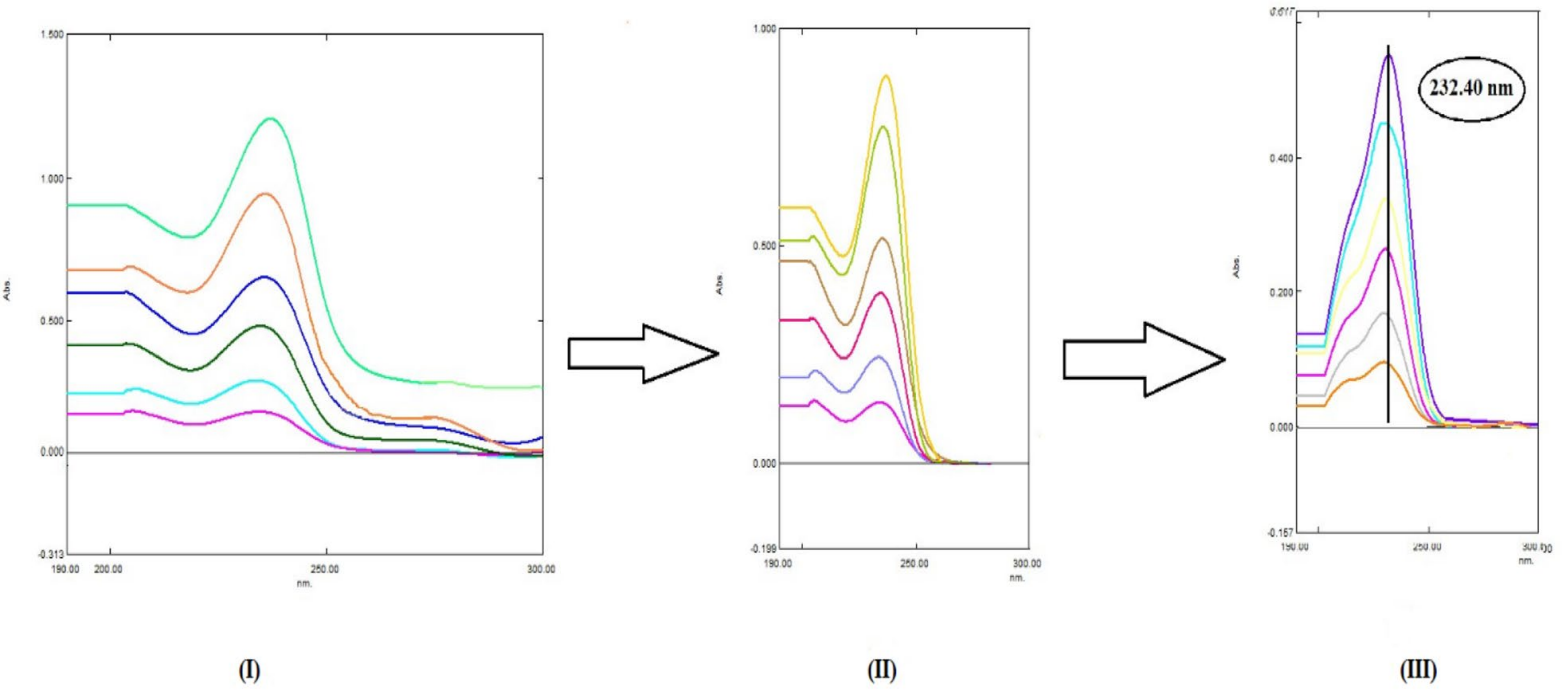
Steps of ratio subtraction method where **I** The ratio spectra of AMC in the range of 5.0–40.0 µg/mL using 20.0 µg/mL DLF as a divisor. **II** The ratio spectra of AMC in the range of 5.0–40.0 µg/mL using 20.0 µg/mL DLF as a divisor after applying subtraction step. **III** The obtained zero order absorption spectra of AMC in the range of 5.0–40.0 µg/mL after multiplication by the same divisor (20.0 µg/mL DLF)

Whereas, AMC can be quantified after removing the effect of the DLF through division of the its spectra and spectra of the synthetic mixtures over a selected concentration of DLF, 20.0 µg/mL, to give a new ratio spectrum. The extension of the obtained spectra was further exploited by subtraction of the absorbance values in plateau region (251.0–400.0 nm). Then, the produced spectra were multiplied by the same divisor (20.0 µg/mL of DLF) to produce the original spectra as represented in Fig. 9. Subsequently, the absorbance at 232.4 nm of the obtained absorption spectra were measured and plotted against its corresponding concentrations and the regression equation was calculated and represented in Table 1.

### Validation

The suggested methods were tested regarding; linearity, range, limit of detection (LOD), limit of quantification (LOQ), selectivity, accuracy and precision and validated according to ICH guidelines [30].

#### Linearity and working range

Under the described experimental conditions, the calibration curves for all spectrophotometric methods were constructed by plotting previously mentioned functions versus each drug concentrations in μg/mL. It was found that all calibration curves were rectilinear over the cited concentration range of 5.0–40.0 μg/mL for both drugs with high values of the correlation coefficients and all regression parameters are supplied in Table 1.

#### Accuracy and precision

The proposed methods were screened for their accuracy by analyzing six different concentrations of each drug within the specified range three times each and the obtained results showed high degree of closeness between the true and measured values and Table 2 shows good percent found of both drugs with low standard deviation values. Moreover, the obtained results of the proposed methods were compared with those obtained by previously reported HPLC method [29] using variance ratio *F*-test and student’s *t*-test [39] indicating that there was no remarkable difference between the proposed methods and comparison one which confirms excellent accuracy. The reported HPLC method discussed that the chromatographic separation was carried out using a reversed phase BDS Hypersil C18 column with a mobile phase consisting of methanol: acetonitrile: water: orthophosphoric acid (60: 30: 9: 1 by volume), at a flow rate of 1 mL /minute and UV detection at 250 nm.

**Table 2:** Application of the proposed univariate spectrophotometric methods for the determination of AMC and DLF in raw materials

Conc. taken (μg/mL)	% Found^a^											
	D^1^		D^2^		RD		DD^1^		RS		Comparison method [29]	
	AMC	DLF	AMC	DLF	AMC	DLF	AMC	DLF	AMC	DLF	AMC	DLF
5.0	101.70	101.06	98.50	100.00	98.84	97.98	99.58	98.54	97.96	101.52	97.95	101.93
10.0	102.08	99.89	98.36	98.21	99.30	100.17	98.77	100.08	98.12	98.30	101.39	100.91
15.0	100.75	98.89	101.69	98.77	101.85	100.35	98.50	98.60	100.40	101.08	100.39	97.98
20.0	100.00	100.96	100.43	100.95	98.47	99.98	100.07	101.96	101.43	100.64	99.56	100.83
30.0	98.36	101.93	101.17	99.36	101.95	101.63	101.85	99.52	100.11	99.10		
40.0	101.89	99.73	99.56	101.94	99.31	99.04	98.69	99.30	99.04	100.59		
Mean ± S.D	100.79 ± 1.43	100.41 ± 1.10	99.95 ± 1.38	99.45 ± 1.06	99.95 ± 1.54	99.85 ± 1.24	99.57 ± 1.26	99.74 ± 1.39	99.51 ± 1.37	100.20 ± 1.24	99.82 ± 1.45	100.41 ± 1.69
t-test^*^	1.04	2.8 × 10^–3^	0.14	0.55	0.13	0.60	0.28	0.80	0.35	0.23		
F-test^*^	1.03	2.36	1.11	1.48	1.12	1.87	1.32	1.80	1.12	1.87		

^a^Each result is the average of three separate determinations

^*^The values of the tabulated t, F at P = 0.05 [39] are 2.30 and 5.40, respectively

Two levels of precision were confirmed using intra and inter-day analysis through application of the proposed methods for triplicate estimation of three concentration levels within the specified range for each drug in the same day (Intra-day precision) and on three different days (Inter-day precision). The % relative standard deviation values were calculated and the results, summarized in Table 3, deducing good precision of the suggested methods.

**Table 3:** precision data for the assay of the studied drugs by the univariate spectrophotometric methods

Parameter	Drug concentration μg/mL	AMC				DLF			
Method		%RSD^a^	%RSD^b^	%Error^a^	%Error^b^	%RSD^a^	%RSD^b^	%Error^a^	%Error^b^
D^1^	10.0	0.777	1.341	0.449	0.774	0.891	0.927	0.514	0.535
	20.0	0.671	1.351	0.387	0.780	0.820	1.146	0.473	0.662
	30.0	0.909	1.597	0.525	0.922	0.619	0.788	0.357	0.455
D^2^	10.0	1.380	1.397	0.797	0.807	0.712	0.806	0.479	0.582
	15.0	0.457	1.140	0.264	0.658	0.945	1.048	0.384	0.427
	20.0	0.922	1.216	0.532	0.702	1.014	1.355	0.308	0.446
RD	5.0	0.797	1.029	0.460	0.594	0.780	1.195	0.450	0.690
	15.0	0.648	1.048	0.374	0.605	0.662	1.191	0.382	0.688
	40.0	0.692	0.891	0.400	0.514	1.160	1.167	0.670	0.674
DD^1^	5.0	1.022	1.350	0.590	0.779	0.700	0.988	0.404	0.570
	15.0	0.881	1.026	0.509	0.592	0.967	0.976	0.558	0.563
	40.0	0.566	0.803	0.327	0.509	0.687	0.993	0.397	0.573
RS	5.0	0.778	0.772	0.446	0.449	0.349	0.542	0.201	0.313
	15.0	0.771	0.972	0.445	0.561	0.729	0.904	0.421	0.522
	40.0	0.398	0.835	0.230	0.482	0.763	0.820	0.441	0.473

^a^^,b^The intra-day and inter-day precision, respectively (n = 3)

#### Selectivity

Selectivity of the proposed methods were inspected through analysis of AMC and DLF in their synthetic mixtures, single and combined pharmaceutical dosage forms without need for prior separation. The obtained good % found affirmed proposed methods selectivity and that there were no interferences from the commercial excipients as represented in Tables 4, 5 and 6.

**Table 4:** Application results for the analysis of AMC and DLF in laboratory synthetic mixtures by the proposed methods

Conc. taken (μg/mL)		% Found^a^											
		D^1^		D^2^		RD		DD^1^		RS		Comparison method [29]	
AMC	DLF	AMC	DLF	AMC	DLF	AMC	DLF	AMC	DLF	AMC	DLF	AMC	DLF
5.0	5.0	100.00	98.87	101.81	100.00	98.24	98.08	100.93	99.18	98.44	99.85	98.66	98.23
10.0	10.0	100.00	100.84	100.90	99.60	99.12	101.07	99.46	100.16	100.38	100.45	101.23	99.27
20.0	20.0	101.35	101.82	99.090	101.00	100.85	100.01	98.38	101.89	101.74	101.10	100.28	101.43
30.0	30.0	100.00	99.96	101.21	99.33	101.59	99.44	101.16	99.67	99.09	100.85	99.54	100.69
Mean ± SD		100.33 ± 0.68	100.37 ± 1.25	100.75 ± 1.17	99.98 ± 0.731	99.95 ± 1.53	99.65 ± 1.24	99.98 ± 1.30	100.22 ± 1.17	99.91 ± 1.45	100.56 ± 0.54	99.93 ± 1.09	99.91 ± 1.43
t-test^*^		0.63	0.42	1.02	0.09	0.02	0.27	0.07	0.34	0.13	0.86		
F-test^*^		2.61	1.30	1.15	3.82	1.98	1.31	1.43	1.47	1.78	9.00		

^a^Each result is the average of three separate determinations

^*^The values of the tabulated t, F at P = 0.05 [39] are 2.44 and 9.27, respectively

**Table 5:** Analysis of AMC and DLF in their single dosage forms; E-Mox® capsules and Declofenac® tablets, respectively

Conc. taken (μg/mL)		% Found^a^											
		D^1^		D^2^		RD		DD^1^		RS		Comparison method [29]	
AMC	DLF	AMC	DLF	AMC	DLF	AMC	DLF	AMC	DLF	AMC	DLF	AMC	DLF
5.0	5.0	100.54	100.74	98.18	100.80	99.27	101.18	100.93	100.00	98.29	100.56	98.12	99.86
10.0	10.0	101.35	99.90	100	98.40	100.15	98.74	98.12	98.79	101.16	98.70	101.23	100.17
20.0	20.0	100.00	99.95	99.09	99.00	98.53	100.04	99.06	99.94	99.80	100.40	100.22	98.38
30.0	30.0	100.90	101.52	100.60	98.66	101.94	99.18	101.61	101.10	100.38	99.33	99.67	101.15
Mean ± S.D		100.69 ± 0.57	100.53 ± 0.77	99.46 ± 1.06	99.21 ± 1.08	99.97 ± 1.46	99.79 ± 1.07	99.93 ± 1.62	99.96 ± 0.94	99.91 ± 1.21	99.75 ± 0.89	99.81 ± 1.29	99.89 ± 1.14
t-test^*^		1.24	0.925	0.414	0.862	0.162	0.130	0.111	0.090	0.107	0.201		
F-test^*^		5.16	2.23	1.49	1.11	1.27	1.13	1.55	1.46	1.14	1.66		

^a^Each result is the average of three separate determinations

^*^The values of the tabulated t, F at P = 0.05 [39] are 2.44 and 9.27, respectively

**Table 6:** Analysis of AMC and DLF in their combined dosage forms Bipen ®capsules

Conc. taken (μg/mL)		% Found^a^											
		D^1^		D^2^		RD		DD^1^		RS		Comparison method [29]	
AMC	DLF	AMC	DLF	AMC	DLF	AMC	DLF	AMC	DLF	AMC	DLF	AMC	DLF
5.0	5.0	101.08	98.87	101.81	101.20	100.30	101.96	99.59	100.39	98.60	101.26	98.66	98.96
10.0	10.0	100.54	98.97	100.90	99.00	99.63	100.29	101.47	99.37	99.61	100.10	101.50	100.63
20.0	20.0	98.64	101.35	99.09	101.00	98.01	99.93	100.40	101.40	100.19	99.87	99.92	98.72
30.0	30.0	99.09	99.96	100.30	100.00	101.76	98.92	98.92	99.80	101.42	100.97	100.08	100.92
Mean ± S.D		99.84 ± 1.15	99.79 ± 1.15	100.53 ± 1.14	100.3 ± 1.01	99.93 ± 1.55	100.28 ± 1.26	100.10 ± 1.09	100.24 ± 0.878	99.95 ± 1.17	100.55 ± 0.667	100.04 ± 1.16	99.81 ± 1.12
t-test^*^		0.248	0.417	0.594	0.644	0.115	0.555	0.069	0.610	0.105	1.13		
F-test^*^		1.01	1.04	1.54	1.23	1.79	1.25	1.11	1.64	1.02	2.85		

^a^Each result is the average of three separate determines

^*^The values of the tabulated t, F at P = 0.05 [39] are 2.44 and 9.27, respectively

#### Sensitivity

Limits of detection (LOD) and quantification (LOQ) for each method were calculated using following ICH equations [30] and their small values for all methods confirmed the good sensitivity as presented in Table 1

- LOD = 3.3 S_a_ /b
- LOQ = 10 S_a_ /b

Where S_a_ is the intercept standard deviation and b is the calibration curve slope.

### Applications

#### Analysis of AMC and DLF in their synthetic mixtures, single and combined dosage forms

The five suggested univariate methods were perfectly applied for estimation of the studied drugs in their synthetic mixtures, single and combined dosage forms. The obtained results were in a good harmonization with those obtained from comparison method [29] as shown in Table 4, 5 and 6. Moreover, the performance of proposed and comparison methods were compared using student’s t-test and variance ratio F-test [39] and their results showed no remarkable differences in terms of accuracy and precision.

### Greenness assessment

The introduction of the green chemistry dimension into the analytical methods assessment should be a natural development trend in chemistry [40]. Most efforts in making analytical methods greener emphasize the need for the elimination of solvents or at least using less toxic, safer and more benign solvents and auxiliaries as well as lowering energy consumption through the dependence on the reactions with mild conditions, avoiding derivatization reactions and preference for substrates based on renewable sources. One of the challenges in green chemistry field is the evaluation of the greenness of chemical processes [41]. Wherefore, the proposed method was assessed by three assessment tools; NEMI, Analytical Eco-scale and GAPI.

NEMI is a one of the oldest methods for assessment of the analytical procedures greenness. The pictogram is a circle consisting of four parts. Each quarter represent different aspect of the described analytical methodology and it colored green if certain requirement is fulfilled. Table 7 shows that the proposed method met all requirements as the used chemicals are not persistent, bio accumulative and toxic and none of the chemicals is listed at hazardous wastes lists and the applied pH during the whole experiment is within the safe range (2.0–12.0) to avoid a highly corrosive environment during the whole analytical process. Finally, the produced waste is less than 50 g [41].

**Table 7:** Evaluation results of the greenness of suggested techniques

(1) Green Analytical Procedure Index (GAPI)	


(2) NEMI pictogram	


(3) Analytical eco-scale score	
Item	Penalty points
1—Reagent	
Methanol	6
2—UV-vis spectrometer	0
3—Occupational hazard	0
4—Waste	3
Total penalty points	9
Analytical eco-scale score	91

Analytical Eco-Scale is another mean to assess of ecological impact of analytical process. Its result represented by score that is calculated by subtracting method penalty points from the basis of 100 (Ideal green analysis). The higher the value (closer to 100) is, the greener analysis will be [41] (Table 7).

GAPI is a new approach for evaluating the green character of an entire analytical methodology from sample collection to final determination. It is applied for providing not only general but also qualitative information. It represented by a specific symbol with five pentagrams, colored mainly from green through yellow to red depicting low, medium to high impact, respectively [42] (Table 7).

### Whiteness assessment

White Analytical Chemistry (WAC) builds upon GAC principles by integrating three key areas: efficiency (red principles), environmental safety (green principles), and practical economics (blue principles). The proposed methods compared with the reported methods regarding whiteness. Subsequently, it was found that the proposed methods were superior to other methods as shown in Table 8 [43].

**Table 8:**
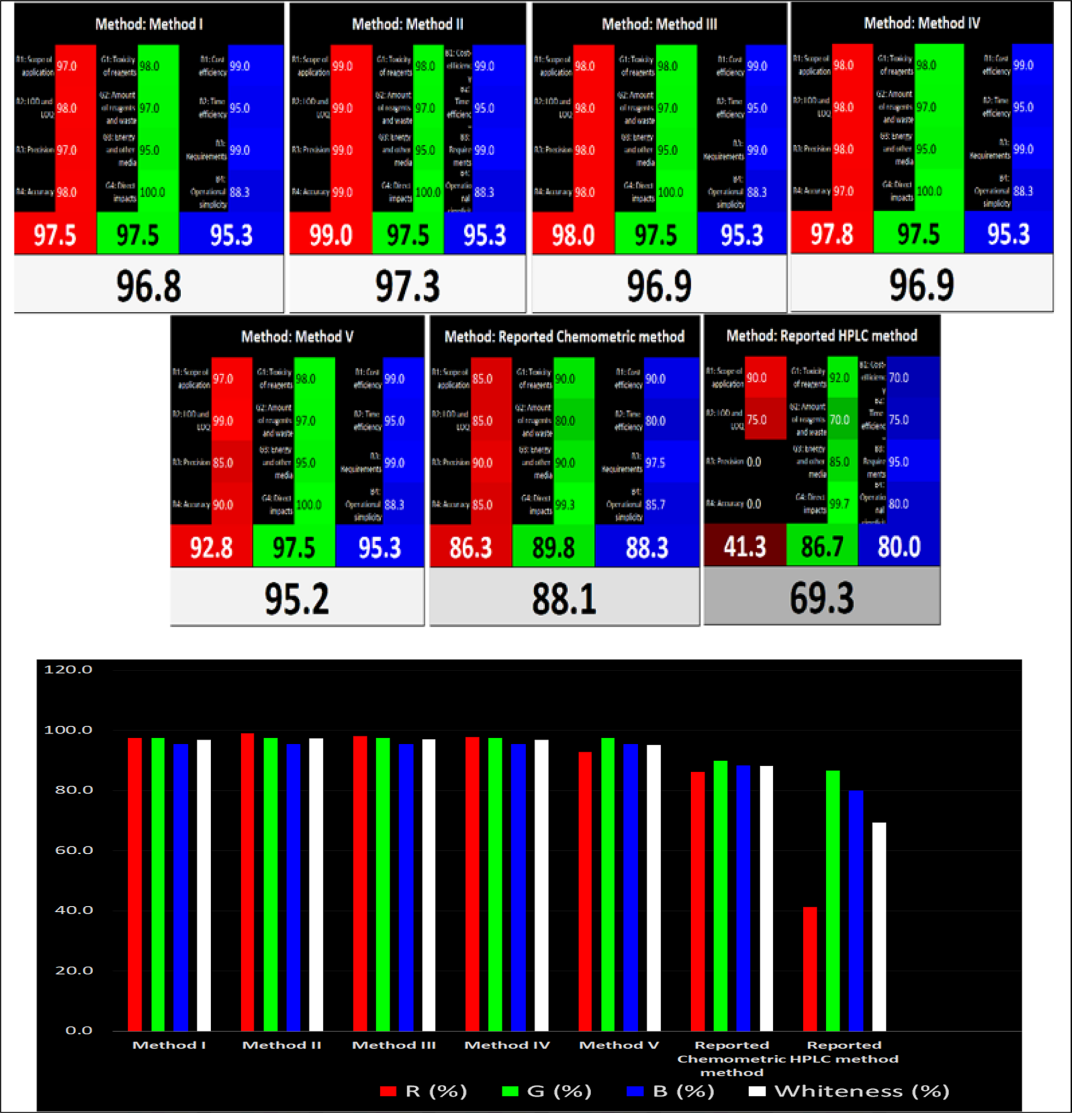
Whiteness of suggested methods and latest reported methods

## Conclusion

The current work represents five univariate spectrophotometric methods for simultaneous estimation of AMC and DLF in their important binary mixture used as one therapy containing anti-biotic and anti-inflammatory in one product. This analysis was performed directly without any extraction or separation steps. The proposed methods are characterized by being rapid, facile, green, cost and effort effective compared to other techniques that require sophisticated steps or instruments. The developed methods were assessed and fully validated according to ICH Guidelines [30]. Furthermore, methods greenness was confirmed through three assessment tools including NEMI, analytical Eco-Scale and GAPI. Therefore, all these merits make the proposed methods worthy for effective application in routine analysis of the studied drugs in their single and combined pharmaceutical preparations.

## Data Availability

The datasets generated during and/or analyzed during the current study are available in the Dryad repository [44]. https://doi.org/10.5061/dryad.70rxwdc5d.
